# Proprotein Convertase Subtilisin/Kexin Type 9 Induces Platelet‐Derived Transforming Growth Factor‐*β* to Promote Myocardial Fibrosis After Myocardial Infarction

**DOI:** 10.1155/humu/4574795

**Published:** 2026-01-21

**Authors:** Qianyun Wang, Wenxiang Huang, Dianmin Xia, Feifei Wang

**Affiliations:** ^1^ Department of Cardiology, The First Affiliated Hospital of Jinan University, Guangzhou, Guangdong, China, jd120.com; ^2^ Health Management Division, The First People’s Hospital of Shunde, Foshan, Gaungdong, China; ^3^ Department of Oncology, The First Affiliated Hospital of Jinan University, Guangzhou, Guangdong, China, jd120.com

**Keywords:** myocardial fibrosis, PCSK9, platelet-derived TGF-*β*

## Abstract

**Aims:**

The recovery of cardiac function after acute myocardial infarction is crucial for the prognosis of patients with myocardial infarction. Proprotein convertase subtilisin/Kexin Type 9 (PCSK9) inhibitors are widely used in patients with acute myocardial infarction due to their potent low‐density lipoprotein‐lowering effects. Recent studies have shown that elevated levels of circulating PCSK9 are associated with increased platelet reactivity and thrombosis; however, the effect and mechanism of PCSK9 on cardiac repair after myocardial infarction through the induction of platelet activation remain unclear. Therefore, the objective of this study was to investigate and clarify the specific effect of PCSK9 on cardiac repair processes following myocardial infarction. The detailed molecular and cellular mechanisms through which PCSK9 regulates cardiac repair after myocardial infarction by inducing platelet activation were observed.

**Methods and Results:**

Hearts from wild‐type (WT) C57BL/6J mice and PCSK9 knockout (PCSK9^−^/^−^) mice were subjected to left coronary artery (LAD) ligation to establish a myocardial infarction model. Six weeks postoperation, echocardiographic analysis and Masson staining revealed that inhibiting the increase in PCSK9 expression after myocardial infarction significantly reduced myocardial fibrosis. Transcriptome sequencing of mouse myocardial tissue suggested that PCSK9 suppresses immune regulation and adhesion pathways and that the platelet marker integrin subunit alpha 2b (Itga2b) is a potential key molecule. Subsequent in vivo and in vitro experiments demonstrated that PCSK9 promotes platelet activation and induces the fibrogenic phenotypic transformation of fibroblasts by transforming growth factor‐*β* (TGF‐*β*). In further studies, coculture experiments of fibroblasts and platelets revealed that PCSK9 promotes the conversion of fibroblasts to myofibroblasts by inducing platelet‐derived TGF‐*β* secretion.

**Conclusion:**

PCSK9 promotes platelet activation, induces the secretion of platelet‐derived TGF‐*β*, and thereby accelerates myocardial fibrosis after myocardial infarction.

## 1. Introduction

Proprotein convertase subtilisin/Kexin Type 9, a liver‐synthesized serineprotease, is crucial for lipid metabolism and atherosclerosis [1]. It degrades LDLR (low‐density lipoprotein receptor), leading to elevated plasma LDL‐C (low‐density lipoprotein cholesterol) levels [2]. In addition, PCSK9 has been implicated in various biological processes, such as the immune response, metabolism, cell cycle, apoptosis, and the inflammatory response [3]. PCSK9 inhibitors have been widely used inclinical practice to reduce LDL‐C levels in high‐risk patients with coronary heart disease [4]. A series of clinical studies have also shown that PCSK9 inhibition can effectively reduce LDL‐C levels and reduce the risk of cardiovascular events in patients with coronary heart disease [5–7]. Our previous studies revealed that PCSK9 affects cardiac function after MI (myocardial infarction) by promoting myocardial fibrosis [8]. The association between PCSK9 and cardiovascular disease is very complex. PCSK9 also regulates autophagy, inflammation, and cellular energy metabolism in the heart [5, 9].

After MI, the number of working cardiomyocytes decreases. The heart subsequently begins to repair and remodel [10]. Myofibroblasts secrete ECM (extracellular matrix) components, including Type I and Type III collagens, elastin, and glycosaminoglycan. At this stage, these ECM components are excessively deposited between cardiomyocytes, leading to cardiac fibrosis. The regulation of myofibroblast proliferation and activity is a key component in the progression of myocardial fibrosis [11, 12]. Previous studies have shown that the overexpression of PCSK9 or stimulation with extracellular recombinant PCSK9 protein enhances the transformation of cardiac fibroblasts into myofibroblasts [13]. This process also increased the protein levels of Type I and Type III collagens and *α*‐SMA. However, the exact mechanism by which PCSK9 affects cardiac fibrosis remains unclear. In our study, we revealed that PCSK9 affects myocardial fibrosis after MI through platelet‐derived TGF‐*β*. This is the first study to propose that PCSK9 affects myocardial fibrosis through the interaction between platelets and cardiac fibroblasts. Platelets play important roles in thrombus formation following atherosclerotic plaque rupture during atherosclerotic exacerbation [14]. After atherosclerotic plaque ruptures, ECM components such as fibronectin, laminin, and collagen are exposed to blood components and release proinflammatory markers and cytokines, resulting in more platelets adhering to the defect site. Adherent platelets further secrete cytoplasmic granules and various chemokines [15]. Previous studies have shown that PCSK9 enhances thrombosis after acute myocardial infarction (AMI) by binding to platelet CD36, leading to increased MI size [2]. However, the role of PCSK9‐induced platelets in myocardial fibrosis remains unclear.

In the present study, we demonstrated that PCSK9 promotes platelet activation and induces platelet‐derived TGF‐*β* secretion and promotes myocardial fibrosis after MI. It is reported for the first time that PCSK9 may participate in the progression of distal cardiac fibrosis by regulating peripheral immune components (platelets).

## 2. Materials and Methods

### 2.1. Animals

C57BL/6 wild‐type mice were obtained from Jiangsu Jicui Yaokang Animal Corp. PCSK9 heterozygous mice on a C57BL/6 background were provided by Lu Xifeng of Shenzhen University, China. All experiments with laboratory animals were approved by the Institutional Ethics Committee of Bojin Biotechnology and complied with the guidelines of the Guide for the Care and Use of Laboratory Animals.

All procedures were conducted following the intraperitoneal administration of 45 mg/kg 2% pentobarbital sodium for anesthesia induction. Specifically, the mice were anesthetized with 2% pentobarbital sodium, after which a small thoracotomy was performed between the fifth and sixth ribs on the left side. The heart was subsequently exposed, and the left anterior descending coronary artery was permanently occluded using 6‐silk thread before the chest was closed. In the sham group, mice underwent identical procedures, except for the ligation of the coronary artery. The mice were euthanized by cervical dislocation under anesthesia for subsequent analysis at 6 weeks postoperation.

#### 2.1.1. Echocardiography

Echocardiography was conducted 6 weeks postoperation to evaluate the left ventricular function of the animals in each group. The mice were anesthetized by 1% isoflurane inhalation, and M‐mode and B‐mode echocardiography were performed using a 12‐MHz probe (Visualsonics Vevo210, Canada). Parameters such as the left ventricular ejection fraction (LVEF), left ventricular fractional shortening (LVFS), and the **E**/**A** ratio were assessed.

#### 2.1.2. EdU (5‐Ethynyl‐2 ^′^‐Deoxyuridine) Incorporation Assay

The EdU incorporation assay was performed using the BeyoClick EdU Cell Proliferation Kit (Beyotime Biotechnology, Cat. No. C0078S) per the manufacturer’s guidelines. Briefly, the cells were incubated with 10 *μ*M EdU for 4 h at 37°C and 5% CO_2_, followed by fixation and permeabilization. After three rinses with PBS, the cells were incubated in Click Additive Solution for 30 min at ambient temperature, and fluorescence imaging was conducted via a confocal laser scanning microscope.

#### 2.1.3. ELISA

PCSK9 levels in peripheral blood were analyzed using a Biolegend LEGEND MAX Mouse PCSK9 ELISA Kit (Fisher Scientific, 50‐207‐9999) according to the provided protocol. A BNP ELISA kit (JL12884‐96T JONLNBIO China) and a TGF‐*β* ELISA kit (JL13959‐96T JONLNBIO China) were used to measure the BNP and TGF‐*β* levels, respectively, in peripheral blood.

#### 2.1.4. Masson Staining

Cardiac tissues were fixed in a 40 g/L paraformaldehyde solution for 48 h, dehydrated through a graded ethanol series, embedded in paraffin, and subsequently sectioned into 4‐*μ*m‐thick pathological slices. Masson staining was performed in accordance with the protocol provided by the Masson staining kit, and cardiac tissue fibrosis was examined under a light microscope [16].

#### 2.1.5. Platelet Preparation

Mouse platelets were extracted as follows: Mice were anesthetized, and 180 *μ*L of blood was aspirated into 20 *μ*L of citrate–glucose solution through the inferior vena cava using a 21‐gauge needle. Platelet‐rich plasma (PRP) was then collected by centrifuging whole blood at 80 × *g* for 10 min. The successfully collected PRP was suspended in two volumes of Tyrode’s solution [17].

#### 2.1.6. Immunohistochemical and Immunofluorescence Staining

Mouse cardiac specimens were fixed in 4% paraformaldehyde for 24–48 h, embedded in paraffin wax, and sectioned. The sections were deparaffinized, treated with ethylenediaminetetraacetic acid (EDTA) solution (pH 9.0), and blocked with 10% goat serum. Primary antibodies (anti‐Smad (1:300)) were applied overnight at 4°C, followed by incubation with secondary antibodies conjugated to horseradish peroxidase. Chromogenic reactions were performed using 3,3 ^′^‐diaminobenzidine, followed by counterstaining with hematoxylin [18]. Immunofluorescence staining involved counterstaining cell nuclei with DAPI (1:1000). Observations were performed using a Leica DM 2500 microscope.

#### 2.1.7. Western Blot (WB) Analysis

Protein from mouse heart, platelet, and cardiomyocyte fibroblasts was prepared with a RIPA Lysis Buffer System (Santa Cruz, CA). A BCA protein assay kit was used to determine the quantity of the protein samples. The proteins were then separated by 10% sodium dodecyl sulfate–polyacrylamide gel electrophoresis (SDS–PAGE) before being transferred to a polyvinylidene fluoride (PVDF) membrane. After blocking with 5% nonfat milk at room temperature for 1 h, the membranes were incubated with primary antibodies against PCSK9 (Ab31762; Abcam, USA), *α*‐SMA (Ab781; Abcam, United States), TGF‐*β* (3711; CST, United States), and *β*‐actin (BM5422; Boster, China) at 4°C overnight. On the second day, the membranes were washed with TBST and then incubated with secondary antibodies (goat anti‐rabbit IgG) for 1 h. Finally, signals were detected using a Bio‐Rad Gel Doc EZ Imaging System (Gel Doc EZ Imager, CA, United States).

#### 2.1.8. Real‐Time Quantitative PCR

RNA was extracted from heart tissue and fibroblasts using TRIzol reagent and reverse transcribed using SuperScript II (Life Technologies, United States) at 42°C. The expression of each gene of interest was measured using SYBR Green PCR core reagents (Applied Biosystems). GAPDH was used as an internal mRNA standard, and the 2^–*ΔΔ*^CT method was used to measure the relative expression levels of each gene. The following primer sequences were used: TGF‐*β* (CGGAGAGCCCTGGATACCA CGGAGAGCCCTGGATACCA), Col1a1 (AGGCGAACAAGGTGACAGAGG GGAGAACCAGGAGAACCAGGAG), Acta2 (GCGTGGCTATTCCTTCGTGACTAC CGTCAGGCAGTTCGTAGCTCTTC), GAPDH (GCAAATTCAACGGCACAGTCAAG TCGCTCCTGGAAGATGGTGATG), Fn1 (CTCGCTTTGACTTCACCACCA TCTCCTTCCTCGCTCAGTTCGTACT), and PCSK9 (AGCAGCCAGGTGGAGGTGTATC GCCTGTCTGTGGAAGCGTGTC).

#### 2.1.9. Transcriptome Analysis

Myocardial samples from the infarcted region were collected from the WT AMI group and the PCSK9‐KO AMI group. Total RNA was extracted using TRIzol reagent following the manufacturer’s instructions. RNA purity and quantification were assessed with a NanoDrop 2000 spectrophotometer (Thermo Scientific, United States), and RNA integrity was evaluated using an Agilent 2100 Bioanalyzer. Transcriptome libraries were prepared according to the protocol of the VAHTS Universal V6 RNA‐seq Library Preparation Kit. Transcriptome sequencing and analysis were performed by Guangzhou Genedenovo Biotechnology Co., Ltd. (Guangzhou, China). Libraries were sequenced on the AB Triple TOF 6600 sequencing platform, and genome alignment was conducted using XCMS software, followed by differential gene expression analysis and calculation of gene expression levels. Principal component analysis (PCA) and gene profile analysis were performed using the gmodels package (v2.18.1) in R to assess the biological reproducibility of the samples. For enrichment analysis based on Gene Ontology (GO) and Kyoto Encyclopedia of Genes and Genomes (KEGG) pathways, a *p* value < 0.05 was considered to indicate statistical significance [19]. Additionally, the interaction relationships from the STRING protein interaction database (http://string-db.org) were used to analyze the protein–protein interaction (PPI) network of the differentially expressed genes [20].

### 2.2. Statistical Analysis

Data from at least three independent experiments are presented as the mean ± SD, and significant differences between two groups were determined using an unpaired *t* test, and one‐way analysis of variance (ANOVA) was used to assess the differences between multiple comparisons, followed by Tukey’s multiple comparisons test. GraphPad Prism was used to perform all the analyses, and a *p* value less than 0.05 was considered to indicate statistical significance.

## 3. Results

### 3.1. Overexpression of PCSK9 After AMI Is Associated With Decreased Cardiac Function and Increased Myocardial Fibrosis in Mice

In this study, 6 weeks after AMI, we performed cardiac color Doppler examination in mice. Compared with those of the sham group, the cardiac function of the mice after AMI decreased significantly (Figure 1a), and the LVEF, LVFS, and *E*/*A* ratio of the mice after MI decreased significantly (Figures 1b, 1c, and 1d) (*p* < 0.0001). ELISA results of peripheral blood revealed that the level of BNP (brain natriuretic peptide) in the AMI group was significantly greater than that in the sham group (Figure 1g) (*p* < 0.001), indicating that heart failure was aggravated after AMI. Moreover, the PCSK9 protein content in peripheral blood increased significantly after AMI (Figure 1h) (*p* < 0.0001). Masson staining was used to evaluate the degree of myocardial fibrosis. The results revealed that myocardial fibrosis was significantly greater in the AMI group than in the sham group (Figure 1e,f) (*p* < 0.0001). The development of fibrosis is related to the uncontrolled accumulation of ECM after MI, and the crucial component is *α*‐SMA. Immunofluorescence detection of *α*‐SMA in the myocardial tissue of the two groups revealed that the content of *α*‐SMA in the myocardial tissue of the MI group was significantly greater than that in the sham group (Figure 1i,j) (*p* < 0.0001). Compared with those in the sham group, the protein and gene expression of PCSK9, *α*‐SMA, and Acta2 (which encodes *α*‐SMA) in the myocardium of the AMI group was significantly upregulated, and the expression of the TGF‐*β* pathway, which is most strongly related to fibrosis, was also significantly upregulated in the myocardium of the AMI group (Figure 1k,l *p* < 0.0001, M *p* < 0.001, N *p* < 0.0001, O *p* < 0.0001, P *p* < 0.0001, and Q *p* < 0.001). These results indicated that cardiac function was impaired, myocardial fibrosis was aggravated, and PCSK9 expression in peripheral blood and myocardial tissue increased significantly after MI.

Figure 1After myocardial infarction, PCSK9 expression increased, cardiac function decreased, and cardiac fibrosis aggravated. (a–d) Compared with the sham and AMI groups, LVEF%, LVFS, and *E*/*A* measured by echocardiography 6 weeks after AMI *n* = 8. (e, f) Masson staining showed significant increase in fibrotic areas (blue) in myocardial tissue of the AMI group. *S* *c* *a* *l* *e* *b* *a* *r* = 100 *μ* *m*, *n* = 5. (g, h) Plasma BNP and plasma PCSK9 were significantly increased in the AMI group *n* = 8. (i) Immunofluorescence staining shows increased *α*‐SMA (green) expression in myocardium of AMI mice, suggesting activation of myofibroblasts, DAPI staining nuclei (blue), *s* *c* *a* *l* *e* *b* *a* *r* = 100 * μ*m. (j) The quantitative analysis of *α*‐SMA immunofluorescence intensity showed that the signal intensity was significantly enhanced in the AMI group *n* = 8. (k–n) Western blot images showed that the expression levels of PCSK9, TGF‐*β*, and *α*‐SMA protein increased in the myocardium of AMI group. Quantitative analysis showed that PCSK9 (l), TGF‐*β* (m), and *α*‐SMA (n) proteins were significantly upregulated in the AMI group *n* = 5. (o‐q) Q‐PCR analysis showed that PCSK9 (o), Acta2 (p), and TGF‐*β* (q) mRNA expressions were significantly upregulated in the myocardium of the AMI group *n* = 5.  ^∗^ *p* < 0.05,  ^∗∗^ *p* < 0.01,  ^∗∗∗^ *p* < 0.001, and  ^∗∗∗∗^ *p* < 0.0001; ns, not significant.

**Figure:**
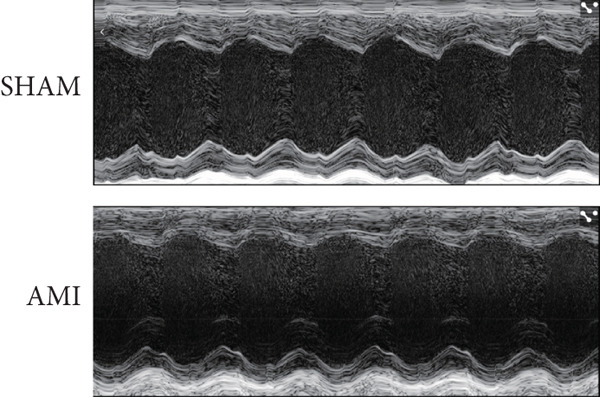
(a)

**Figure:**
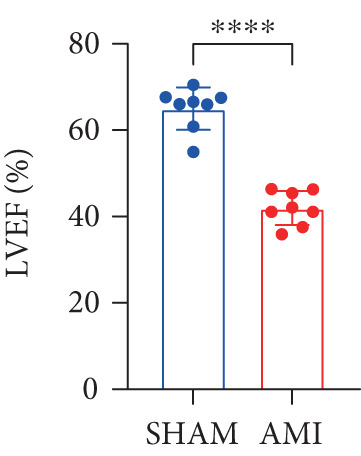
(b)

**Figure:**
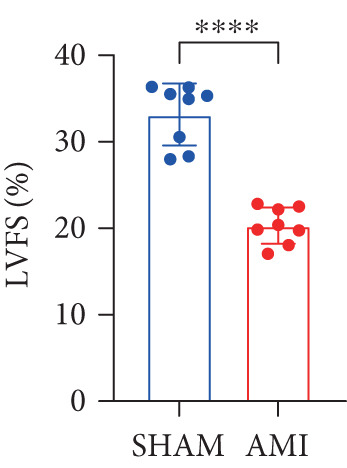
(c)

**Figure:**
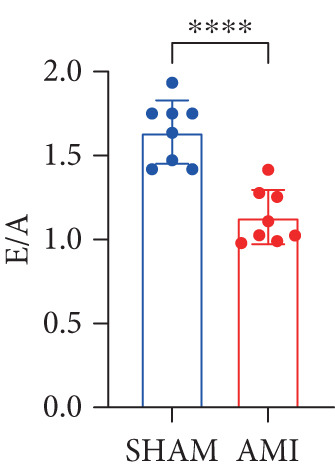
(d)

**Figure:**
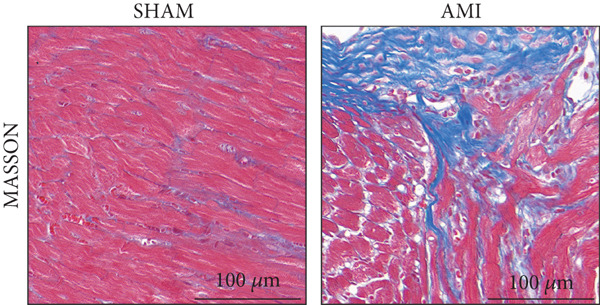
(e)

**Figure:**
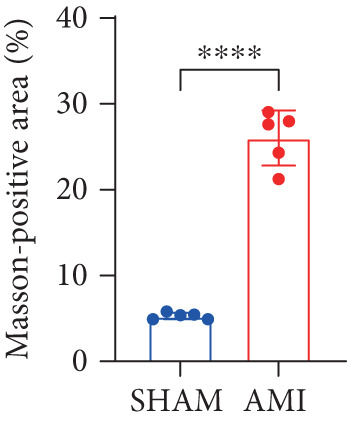
(f)

**Figure:**
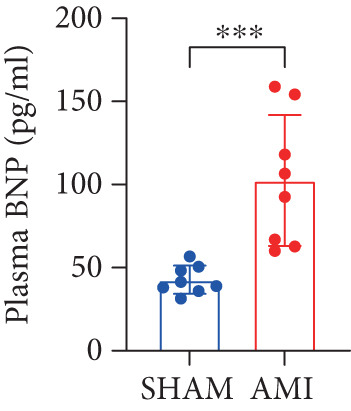
(g)

**Figure:**
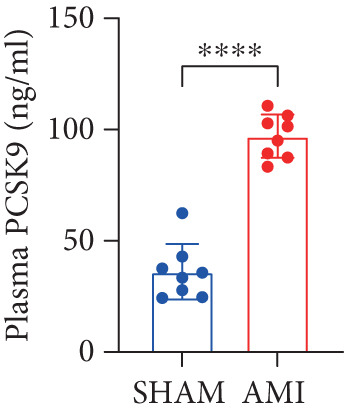
(h)

**Figure:**
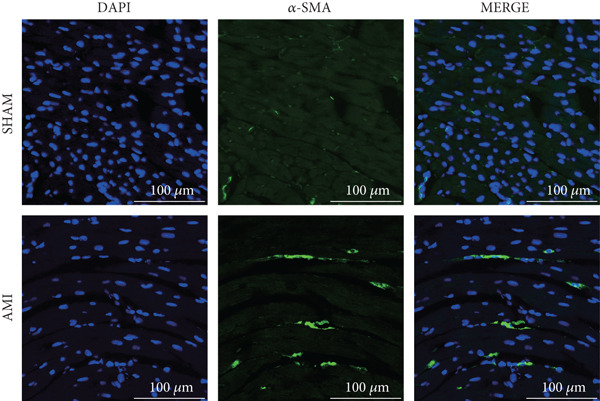
(i)

**Figure:**
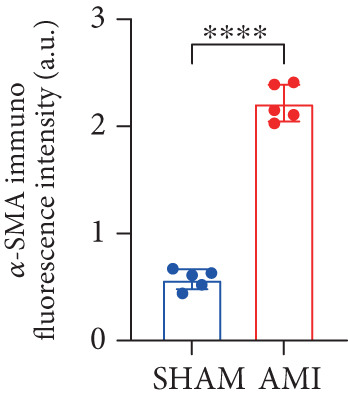
(j)

**Figure:**
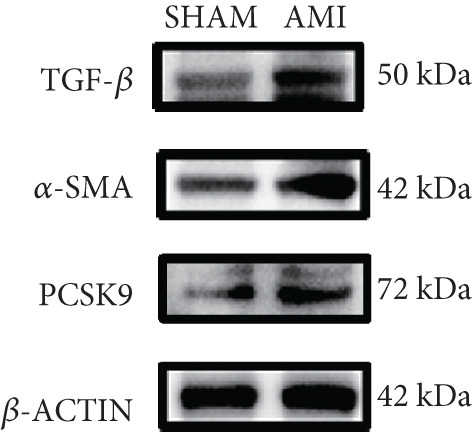
(k)

**Figure:**
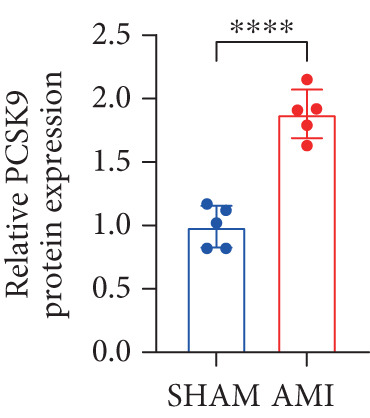
(l)

**Figure:**
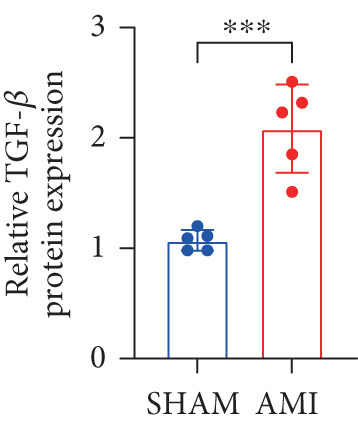
(m)

**Figure:**
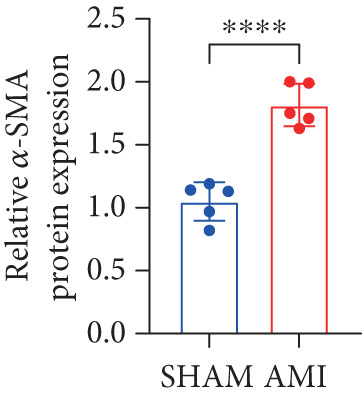
(n)

**Figure:**
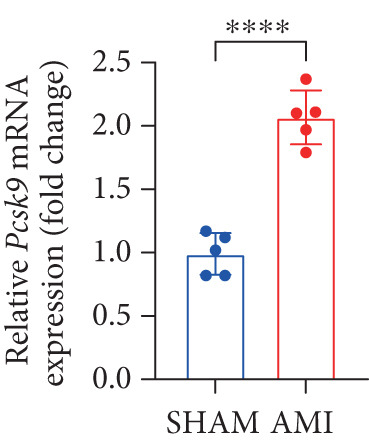
(o)

**Figure:**
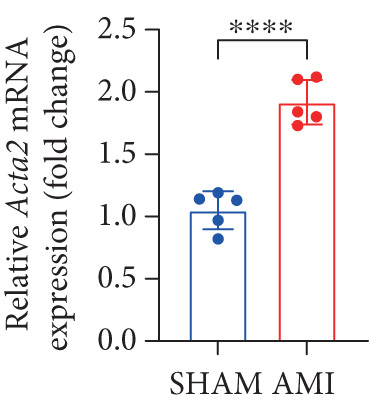
(p)

**Figure:**
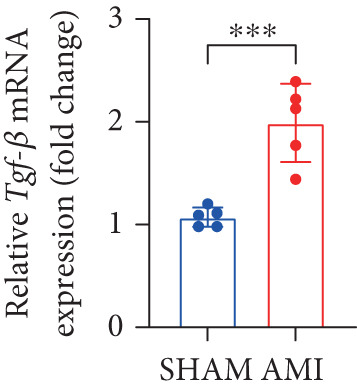
(q)

### 3.2. Inhibition of PCSK9 Expression in Postinfarction Myocardium Suppresses Myocardial Fibrosis, and This Process Is Associated With theTGF‐*β* Signaling Pathway

To investigate the role of PCSK9 in myocardial fibrosis after AMI, we constructed PCSK9 knockout (KO) mice. Cardiac function was evaluated by echocardiography in mice after MI, and the results revealed that inhibition of PCSK9 expression after MI improved cardiac function (Figure 2a). Compared with those in the WT AMI group, the LVEF, LVFS, and *E*/*A* ratio in the PCSK9‐KO AMI group significantly improved (Figure 2b, *p* < 0.05; Figure 2c, *p* < 0.05; and Figure 2d, *p* < 0.0001). The degree of myocardial fibrosis was evaluated via Masson’s trichrome staining, which revealed that myocardial fibrosis was significantly reduced in PCSK9‐KO mice after AMI compared with that in the WT AMI group (Figure 2e,f *p* < 0.0001). Immunofluorescence, WB, and PCR analyses also revealed that the protein and gene expression levels of *α*‐SMA and TGF‐*β* in the myocardium of the PCSK9‐KO AMI group were significantly lower than those in the WT AMI group. These findings further indicate that inhibiting the high expression of PCSK9 after AMI can alleviate myocardial fibrosis and that this process is associated with the TGF‐*β* signaling pathway (Figure 2g,h *p* < 0.0001; Figure 2i,j *p* < 0.01; Figure 2k *p* < 0.001; Figure 2l *p* <0.05; and Figure 2m *p* < 0.05).

Figure 2PCSK9 knockout ameliorates cardiac dysfunction and fibrosis phenotype in mice after myocardial infarction. (a–d) Echocardiographic M‐mode images and quantitative results showed that LVEF, LVFS and E/A decreased significantly in the WT AMI group, while those in KO AMI group were significantly improved compared with the WT AMI group *n* = 8. (e, f) Masson staining and quantitative results showed that collagen deposition (blue) was significantly enhanced in the WT AMI group, while collagen deposition was significantly relieved in the KO AMI group. *S* *c* *a* *l* *e* *b* *a* *r* = 100 * μ*m, *n* = 5. (g, h) Immunofluorescence images showed *α*‐SMA (green) was significantly expressed in the WT AMI group, whereas expression levels decreased in the KO AMI group; DAPI (blue). *S* *c* *a* *l* *e* *b* *a* *r* = 100 * μ*m. Quantitative analysis of *α*‐SMA immunofluorescence intensity showed that the signal intensity of the WT AMI group was the strongest, and the KO AMI group was significantly weaker than the WT AMI group *n* = 5. (i–k) Western blot images and quantitative analysis results showed that the protein expression levels of *α*‐SMA and TGF‐*β* in the KO AMI group were significantly lower than those in the WT AMI group *n* = 5. (l, m) qPCR analysis showed that the expression of Acta2 and TGF‐*β* was upregulated in the WT AMI group, but significantly lower in the KO AMI group than in the WT AMI group *n* = 5.  ^∗^ *p* < 0.05,  ^∗∗^ *p* < 0.01, and  ^∗∗∗^ *p* < 0.001; ns, not significant.

**Figure:**
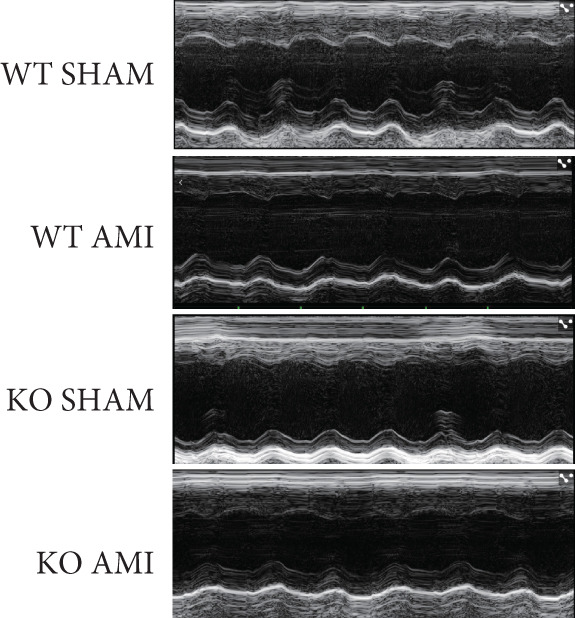
(a)

**Figure:**
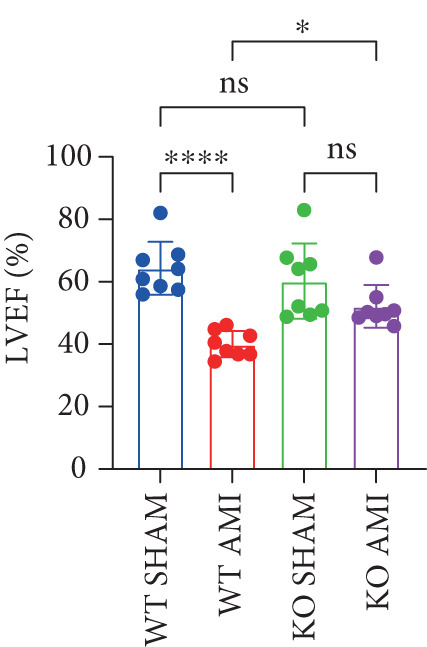
(b)

**Figure:**
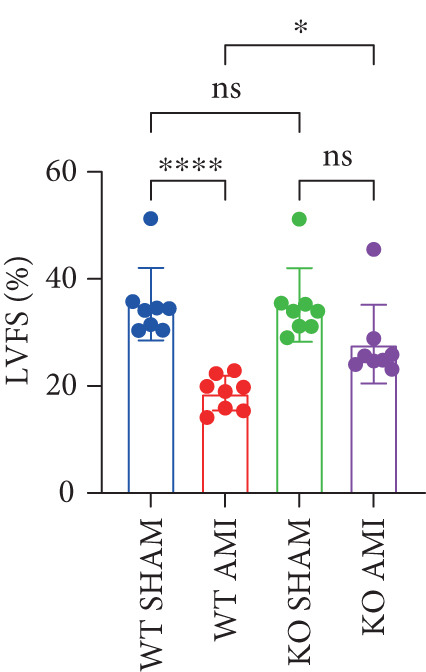
(c)

**Figure:**
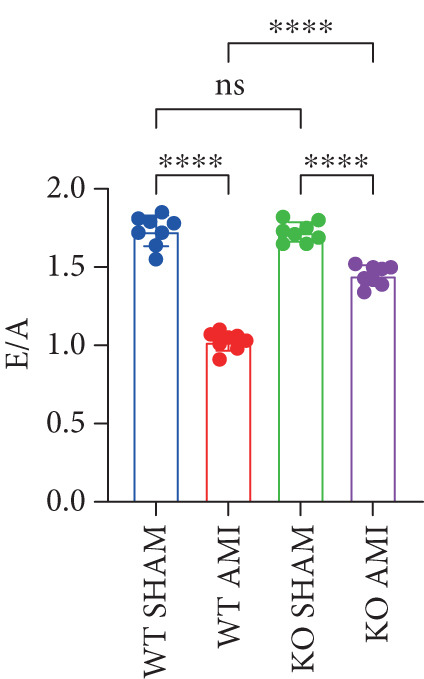
(d)

**Figure:**
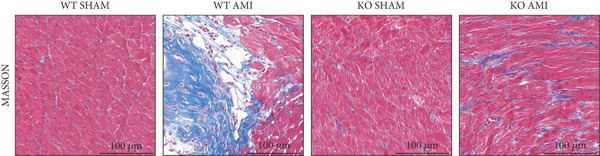
(e)

**Figure:**
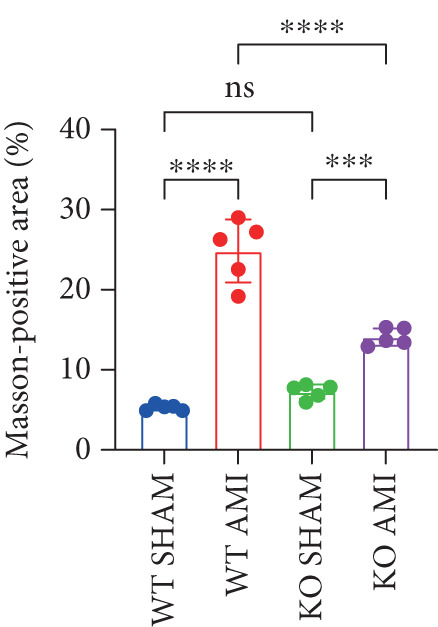
(f)

**Figure:**
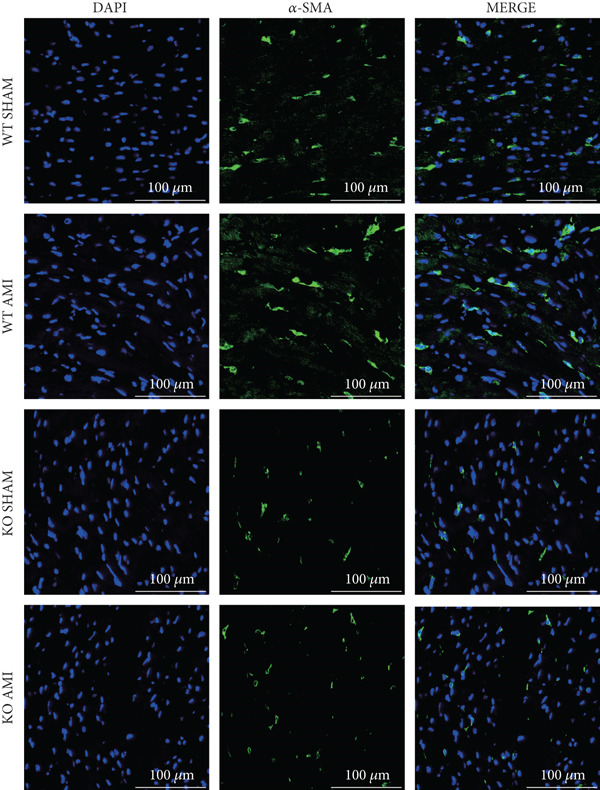
(g)

**Figure:**
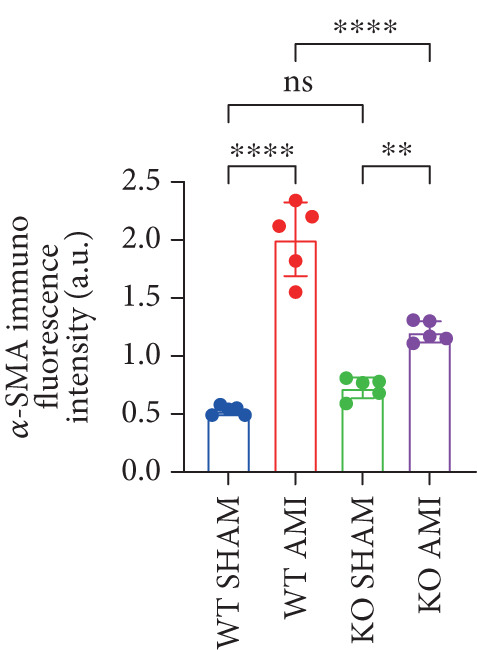
(h)

**Figure:**
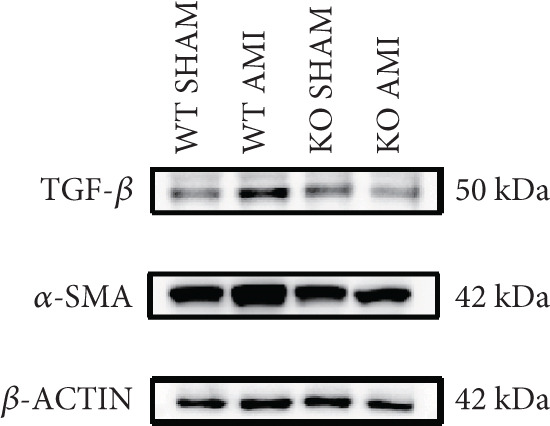
(i)

**Figure:**
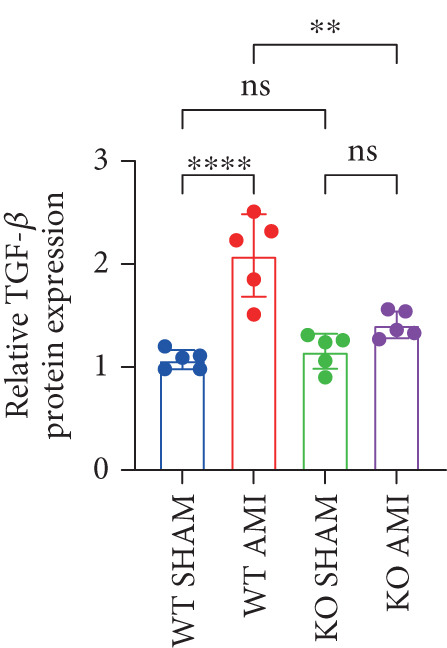
(j)

**Figure:**
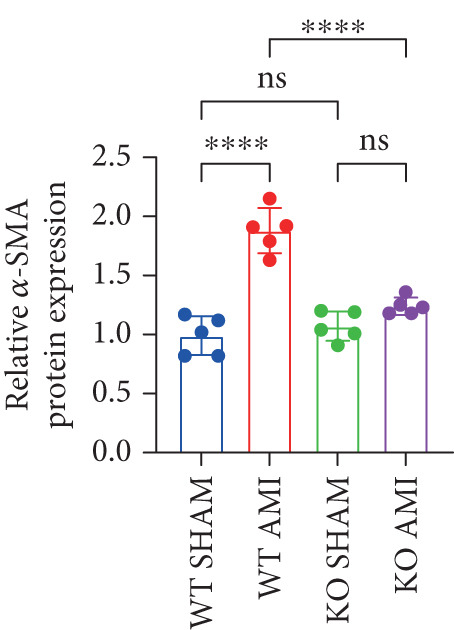
(k)

**Figure:**
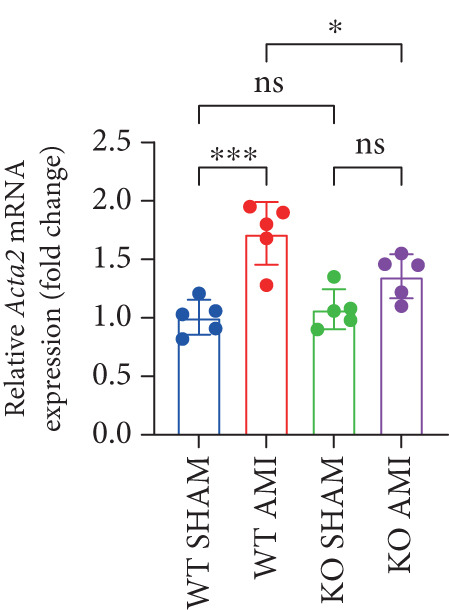
(l)

**Figure:**
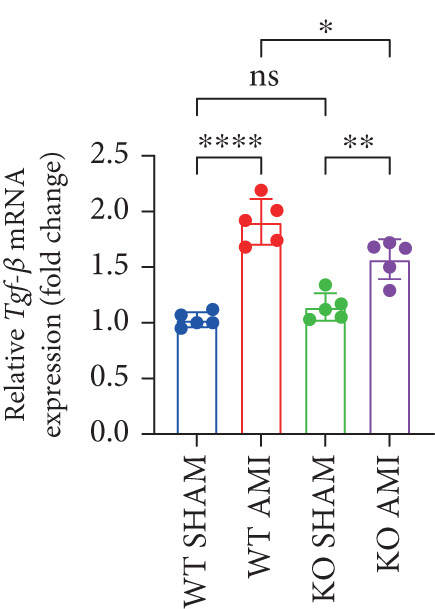
(m)

### 3.3. Transcriptome Analysis Revealed That PCSK9 Deletion Affects Immune Regulation and Adhesion Pathways and That Itga2b Is a Potential Key Molecule

To further clarify the mechanism by which PCSK9 regulates myocardial fibrosis after MI, we performed a sequencing analysis on myocardial tissues from both groups of mice. Volcano plot showing the distribution of genes that were differentially expressed between the two groups (WT AMI vs. KO AMI) in the MI model. Red dots represent upregulated genes, blue dots represent downregulated genes, and gray dots represent genes whose expression did not significantly differ. The threshold was set to |log_2_FC| > 1 and FDR < 0.05. Compared with those in the PCSK9‐KO AMI group, 41 genes whose expression was downregulated and 33 genes whose expression was upregulated in the WT AMI group (Figure 3a).

Figure 3Transcriptome analysis revealed that PCSK9 deletion affects immune regulation and adhesion pathways, and Itga2b is a potential key molecule. (a) Volcano map showing the distribution of differentially expressed genes between the WT AMI group and KO AMI group. (b) The heat map shows the clustering results of expression profiles of differentially expressed genes in each sample. (c) GO enrichment ring plots further synthesize the classification of significant pathways. (d) KEGG enrichment bubble plot is plotted by using the first 20 pathways with the smallest *Q* value. The ordinate is the pathway, and the abscissa is the enrichment factor. The size indicates the number. The redder the color, the smaller the *Q* value. (e) PPI (protein–protein interaction) network constructed based on the differential expression results showed several key immune and adhesion‐related nodes, among which Itga2b was significantly downregulated and located in the center of the network, suggesting that it may be involved in the regulation of platelet activation restriction and immune‐fibrosis axis in PCSK9 deletion state.

**Figure:**
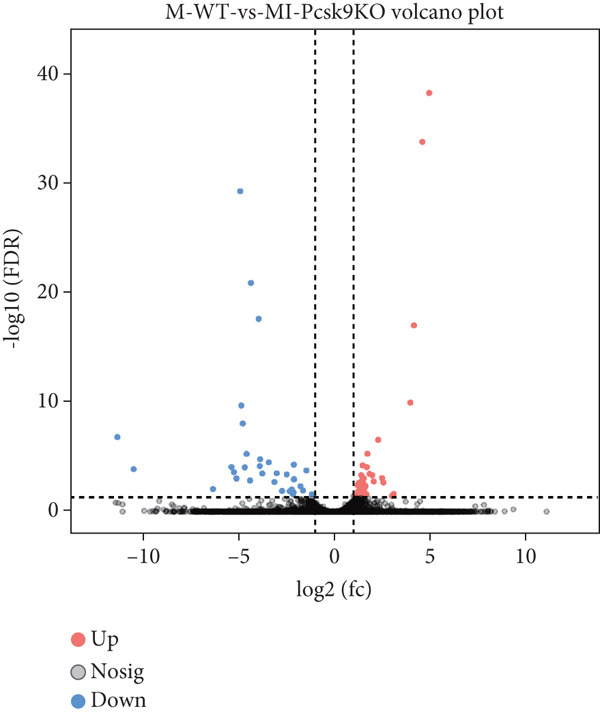
(a)

**Figure:**
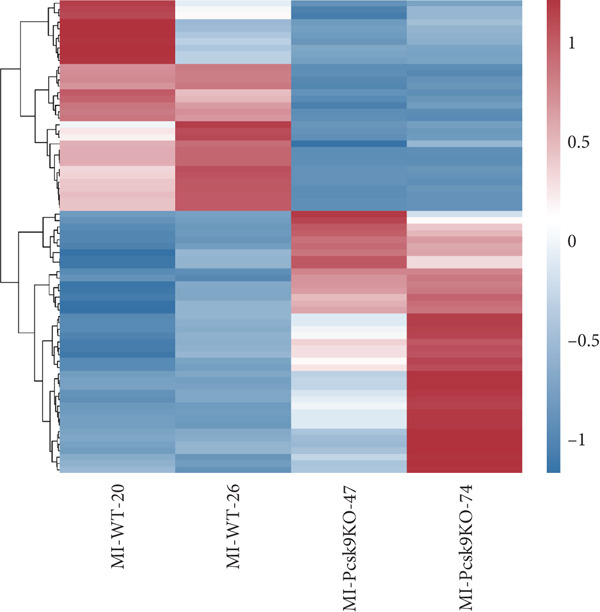
(b)

**Figure:**
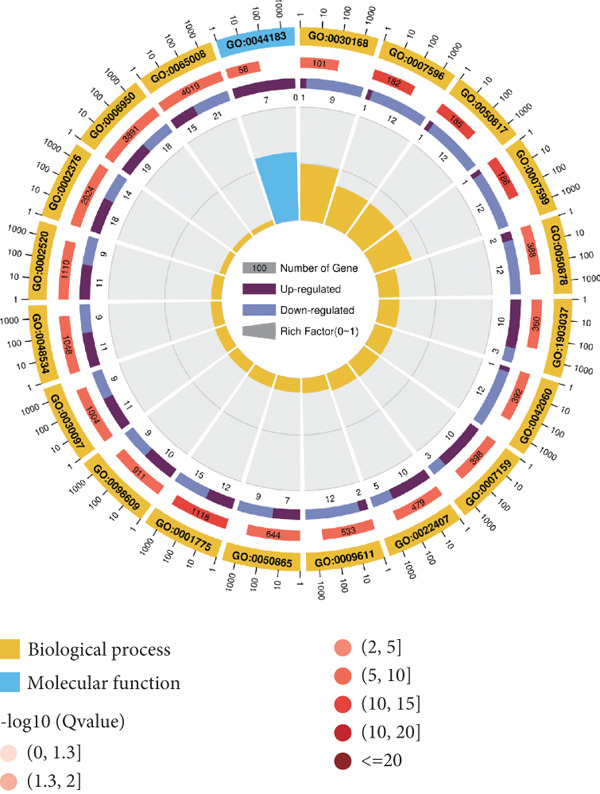
(c)

**Figure:**
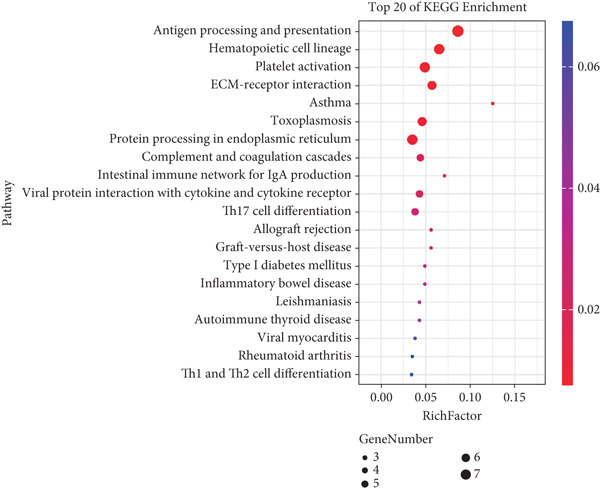
(d)

**Figure:**
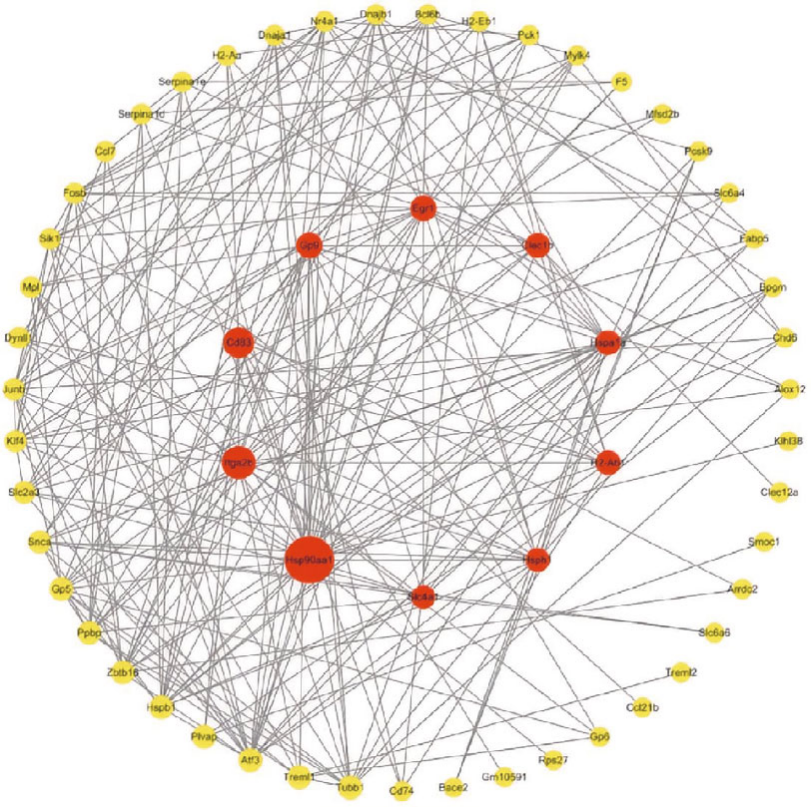
(e)

The heat map shows the clustering results of the expression profiles of differentially expressed genes in each sample, with colors indicating normalized expression levels, reflecting group consistency and differences in expression trends (Figure 3b). The GO enrichment ring plot further comprehensively shows the classification of significant pathways (yellow: biological process and blue: molecular function), the number of upregulated genes, enrichment significance (red color scale, −log *q* value), and enrichment factors (rich factor). It systematically revealed that immune activation and adhesion pathways were widely regulated (Figure 3c). The results of the KEGG enrichment analysis revealed that PCSK9 knockdown was associated mainly with platelet activity and impairment of ECM activation signaling pathways, including antigen processing and presentation, ECM‐recept to interaction, and platelet activation (Figure 3d). The PPI network constructed on the basis of the above differential expression results revealed multiple key immune‐ and adhesion‐related nodes. Among these genes, Itga2b was significantly downregulated and located at the center of the network, suggesting that it might be involved in regulating the restriction of platelet activation and the immune‐fibrosis axis in the state of PCSK9 deletion (Figure 3e). RNA‐seq analysis and systems biology methods revealed changes in the core regulatory pathways caused by PCSK9 deletion, particularly suggesting that Itga2b might be a key molecule mediating PCSK9‐regulated platelet–cardiac interstitial communication, thus establishing an important theoretical basis for subsequent mechanistic experiments.

### 3.4. PCSK9 Promotes Platelet Activation in Post‐MI Mice and Mediates Fibroblast Profibrotic Phenotypic Transformation via TGF‐*β*

To further verify that PCSK9 promotes platelet activation in mice after MI and mediates the fibrogenic phenotypic transformation of fibroblasts via TGF‐*β*, we measured the TGF‐*β* levels, mean platelet volume (MPV), platelet volume distribution width (PDW), and large platelet ratio (P‐LCR) in the peripheral blood of the four groups of mice; this was done to determine whether PCSK9 gene KO affects peripheral blood platelet activation after MI. The results revealed that the plasma TGF‐*β* level was significantly increased in the WT AMI group but significantly decreased in the KO AMI group (Figure 4a, *p* < 0.0001). Further analysis of platelet counts revealed no significant difference in platelet count among the groups (Figure 4b, *p* > 0.05). The MPV significantly increased in the WT AMI group (Figure 4c) (*p* < 0.0001), whereas the PDW did not significantly differ between the WT AMI group and the KO AMI group (Figure 4d) (*p* > 0.05). The proportion of large platelets (P‐LCR) significantly increased in the WT AMI group but decreased in the KO AMI group (Figure 4e) (*p* < 0.05), suggesting that PCSK9 KO can alleviate the trend of platelet activation. As platelet agonists, ADP and thrombin can bind to platelet surface receptors, triggering the release of ADP from inside platelets, which further leads to platelet aggregation. We used ADP and thrombin to stimulate platelets in vitro to assess their activation. Optical density curves revealed that PCSK9 stimulation increased ADP‐ and thrombin‐induced platelet aggregation (Figure 4f,g), increased the maximal aggregation rate (Figure 4h, *p* < 0.001), and shortened the half‐maximal aggregation time (T½) (Figure 4i, *p* < 0.01). Flow cytometry revealed increased expression of integrin *α*IIb (Itga2b) in platelets stimulated with PCSK9 (Figure 4j).

Figure 4PCSK9 promotes platelet activation and TGF‐*β* expression in mice after myocardial infarction. (a) Comparison of TGF‐*β* content in serum of mice in each group *n* = 8. (b–e) Comparison of platelet parameters in each group, (b) the total platelet count among the four groups, (c) MPV increased significantly in the WT AMI group, (d) PDW significantly increased in the WT AMI group and decreased in the KO AMI group, and (e) P‐LCR significantly increased in the WT AMI group and decreased in the KO AMI group *n* = 8. (f–i) PCSK9 stimulates (f, g) platelet aggregation and activation markers’ expression in vitro, (h) maximum aggregation rate increases, and (i) shortened half‐maximum aggregation time (T½). (j) Flow cytometry showed that platelet integrin *α*IIb (Itga2b) expression increased after PCSK9 stimulation.  ^∗^ *p* < 0.05,  ^∗∗^ *p* < 0.01,  ^∗∗∗^ *p* < 0.001, and  ^∗∗∗∗^ *p* < 0.0001; ns, not significant.

**Figure:**
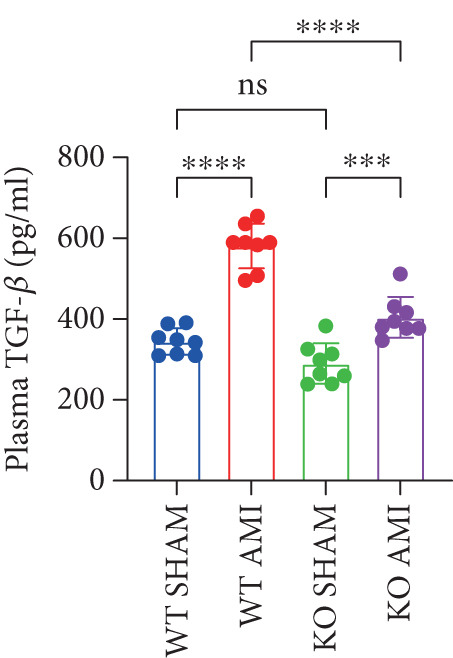
(a)

**Figure:**
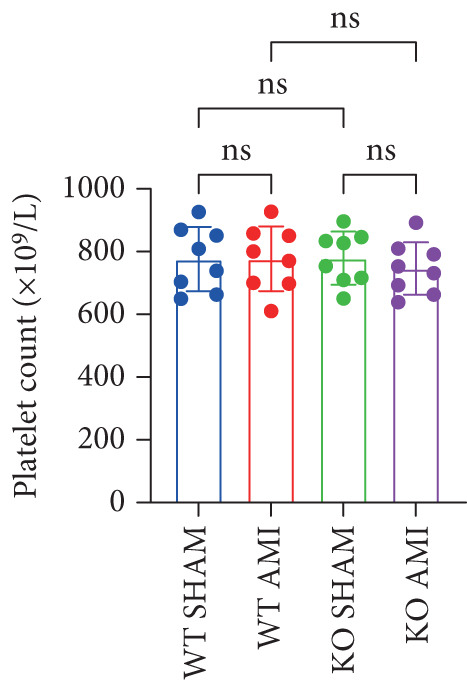
(b)

**Figure:**
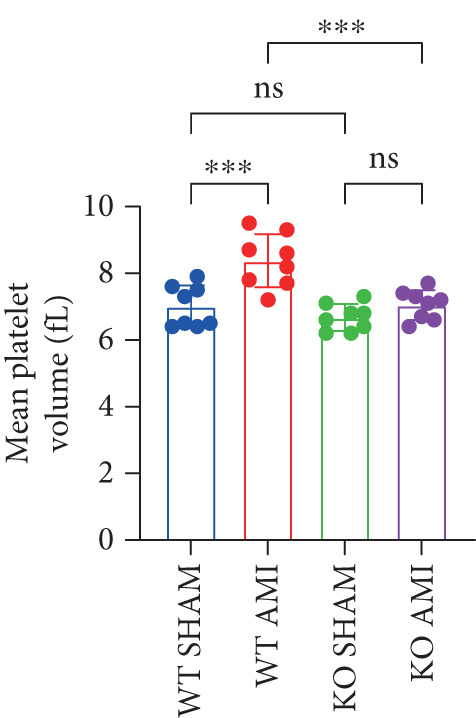
(c)

**Figure:**
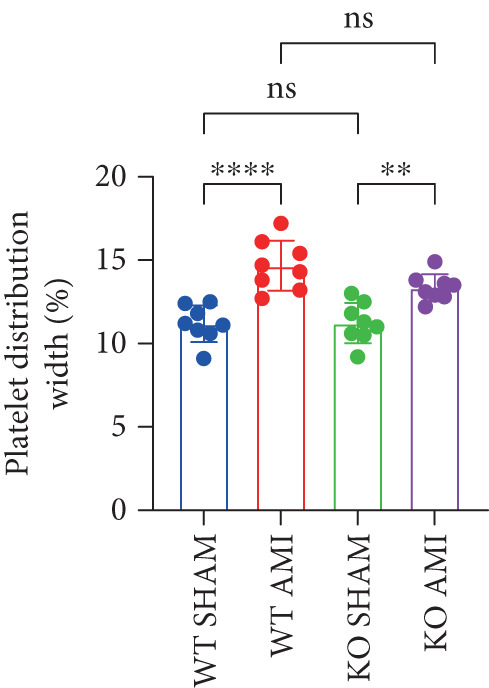
(d)

**Figure:**
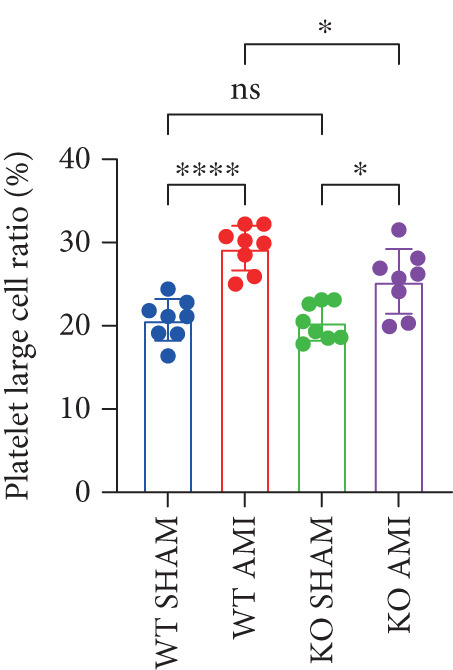
(e)

**Figure:**
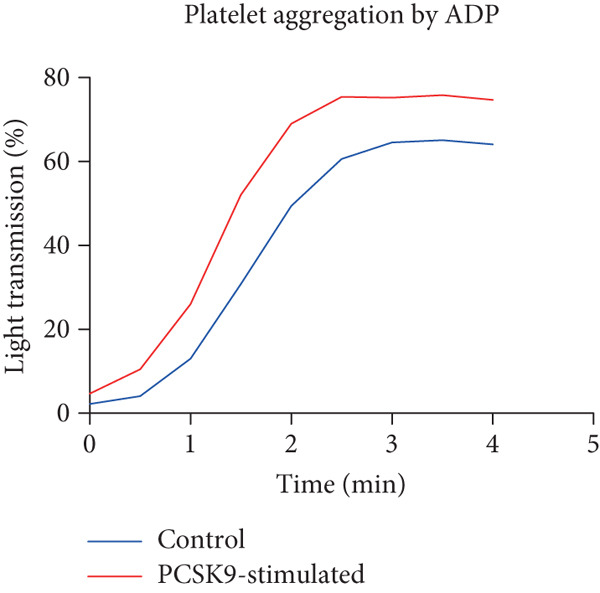
(f)

**Figure:**
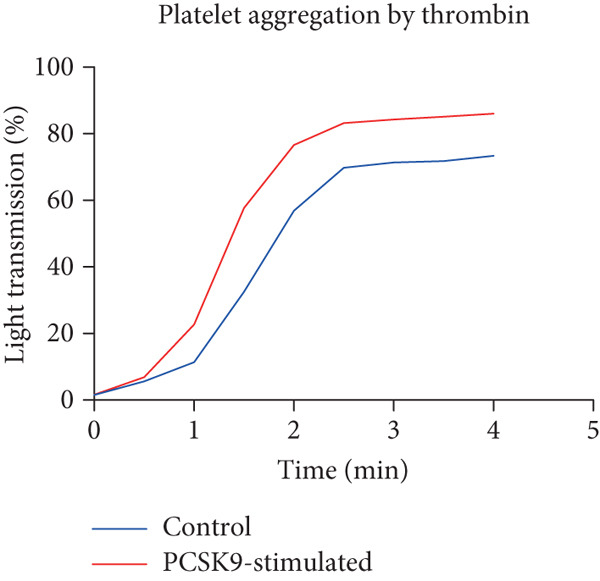
(g)

**Figure:**
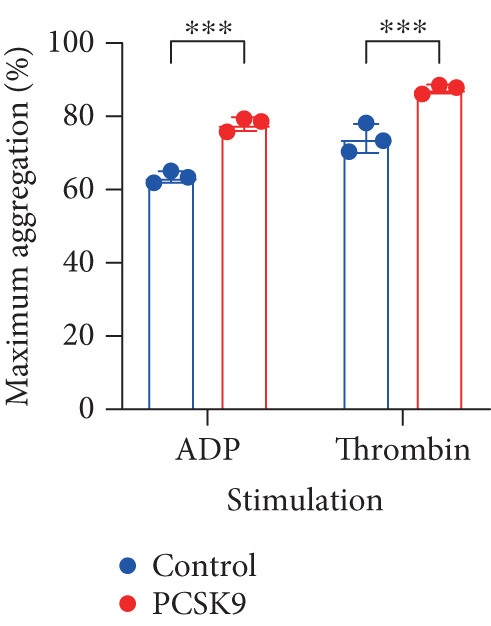
(h)

**Figure:**
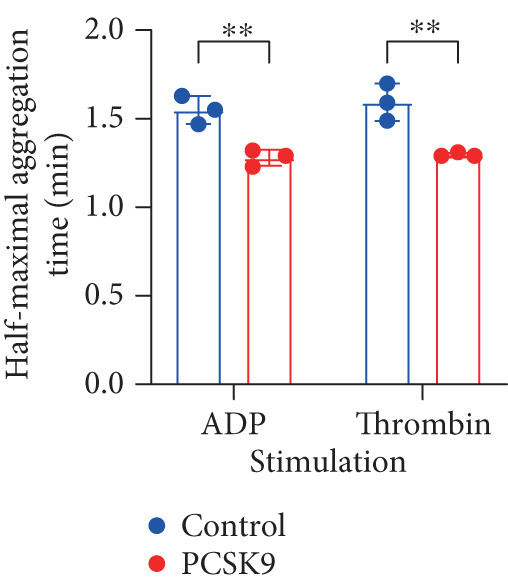
(i)

**Figure:**
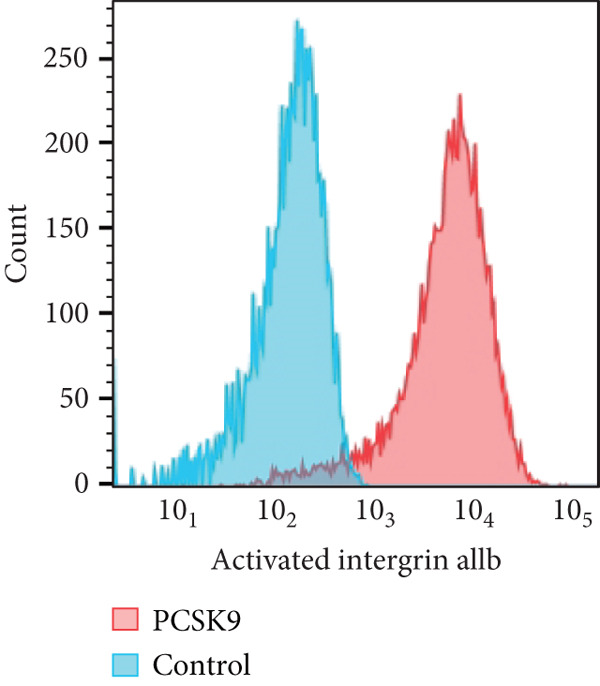
(j)

### 3.5. PCSK9 Induces Platelet‐Derived TGF‐*β* to Promote the Conversion of Fibroblasts to Myofibroblasts

In the previous section, we demonstrated that PCSK9 promotes myocardial fibrosis via platelet activation; however, the underlying pathway and mechanism remain unclear. Therefore, we designed a coculture system of fibroblasts and platelets to further clarify that PCSK9 promotes the conversion of fibroblasts to myofibroblasts by inducing the expression of platelet‐derived TGF‐*β*.

We established five groups: (1) fibroblasts alone; (2) fibroblasts cocultured with platelets; (3) fibroblasts cocultured with platelet supernatant supplemented with the PCSK9 protein; (4) a Transwell coculture system in which the PCSK9 protein was added to the platelet supernatant (upper chamber) and a TGF‐*β* neutralizing antibody was added to the fibroblasts (lower chamber); and (5) fibroblasts treated directly with a TGF‐*β*‐neutralizing antibody.

First, we detected TGF‐*β* in the supernatant of the cells in the PCSK9‐platelet group via ELISA. The results revealed that TGF‐*β* secretion was significantly increased in the PCSK9‐treated group and significantly decreased after the addition of a TGF‐*β*‐neutralizing antibody (Figure 5a, *p* < 0.0001). We further assessed the proliferation and migration of fibroblasts in each group using a EdU assay and a scratch test. PCSK9 treatment significantly increased the proliferation and migration of fibroblasts (Figure 5b,c; *p* < 0.05). After the addition of a TGF‐*β*‐neutralizing antibody, both horizontal migration and proliferation decreased, suggesting that PCSK9 promotes fibroblast migration and proliferation by increasing the level of platelet‐derived TGF‐*β* (Figure 5b,e; *p* < 0.0001).

Figure 5In vitro coculture experiment to verify the fibrogenic effect of PCSK9‐treated platelets. ELISA showed that TGF‐*β* secretion level in supernatant of PCSK9‐platelet group was significantly increased (*n* = 8). (b, c) EdU test and quantitative analysis detected the difference of CF increment ability among groups *n* = 3. (d, e) Scratchhealing test and quantitative analysis were used to detect the difference of CF migration ability among the groups *n* = 3. (f, g) Immunofluorescence staining images and quantitative analysis further showed that PCSK9‐platelet could induce a significant increase in *α*‐SMA expression in fibroblasts, TGF‐*β* blockade could partially reverse this phenotypic transformation, and recombinant TGF‐*β* treatment group showed the highest expression level. Green is VIMENTIN, red is *α*‐SMA, blue is DAPI, *s* *c* *a* *l* *e* = 100 * μ*m. (h–j) Fibrosis‐related genes Col1a1, Fn1, and Acta2 mRNA expression were significantly upregulated in fibroblasts, and TGF‐*β*‐neutralizing antibody treatment could effectively inhibit this trend, and TGF‐*β*‐positive control group showed the strongest expression.  ^∗^ *p* < 0.05,  ^∗∗^ *p* < 0.01,  ^∗∗∗^ *p* < 0.001, and  ^∗∗∗∗^ *p* < 0.0001; ns, not significant.

**Figure:**
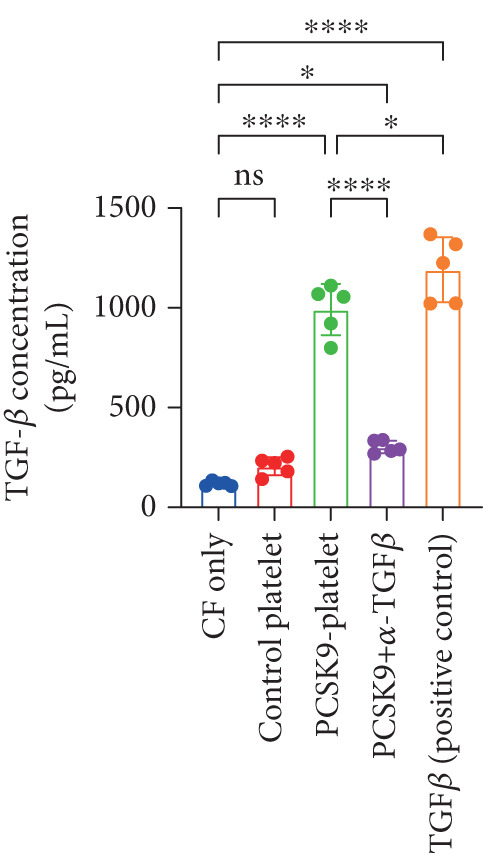
(a)

**Figure:**
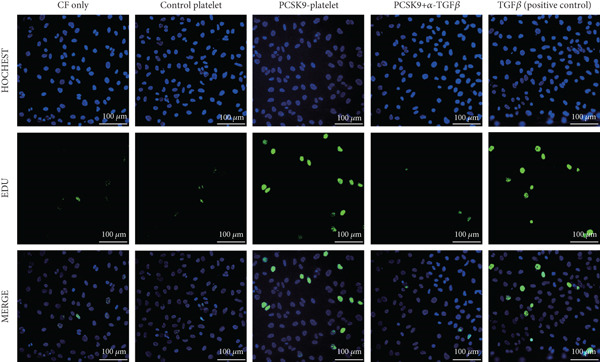
(b)

**Figure:**
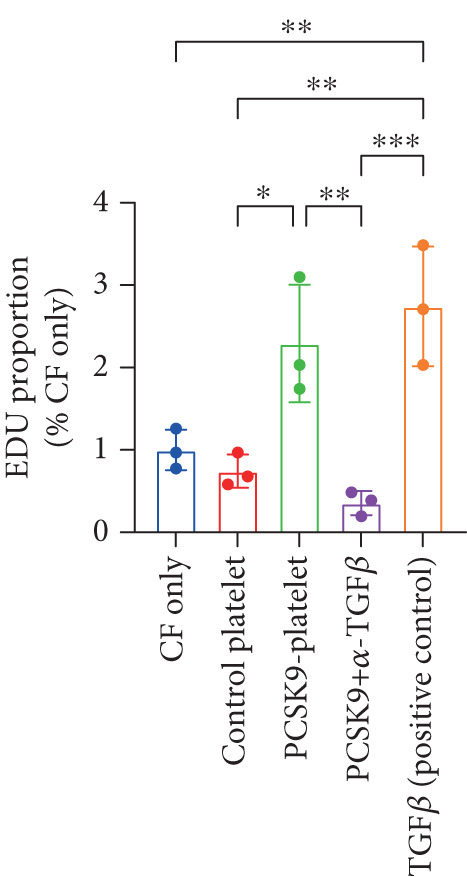
(c)

**Figure:**
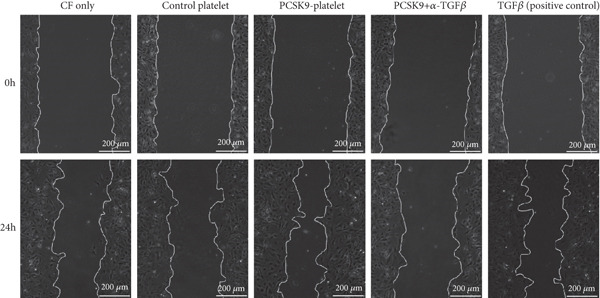
(d)

**Figure:**
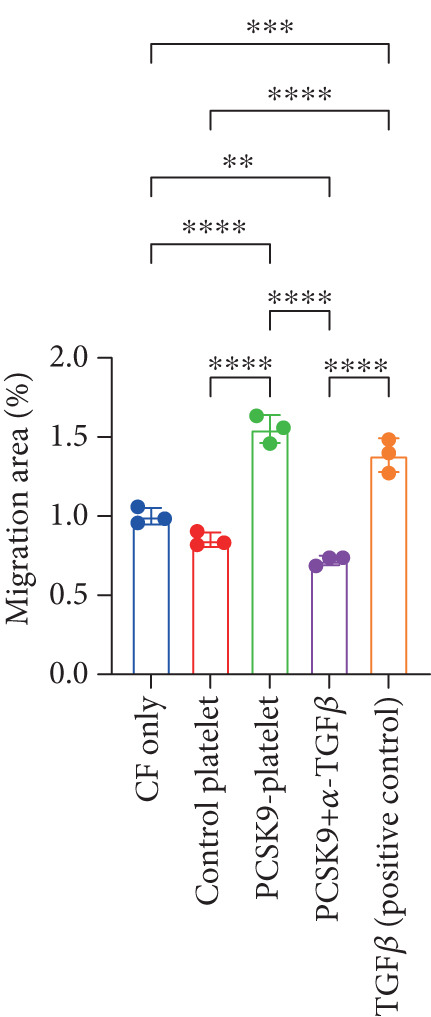
(e)

**Figure:**
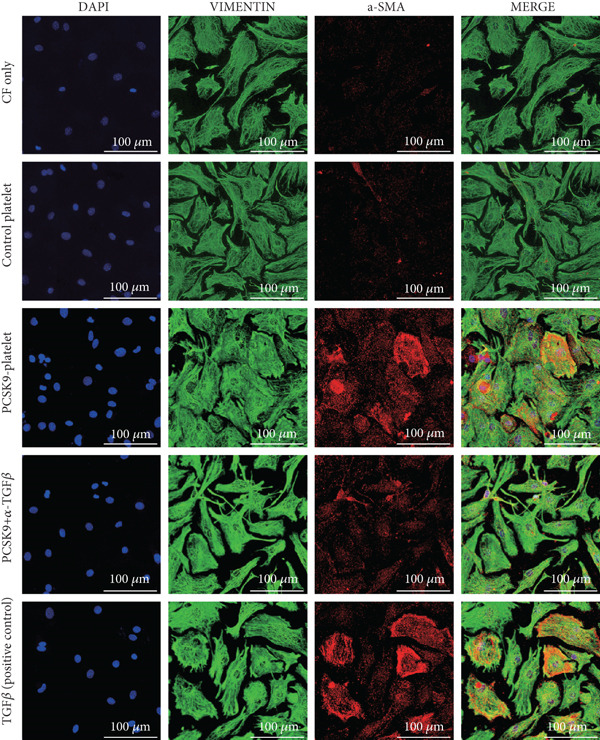
(f)

**Figure:**
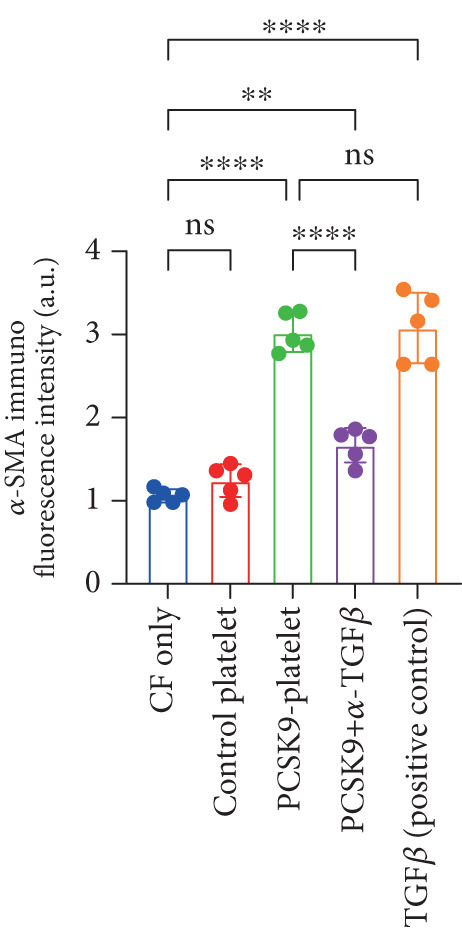
(g)

**Figure:**
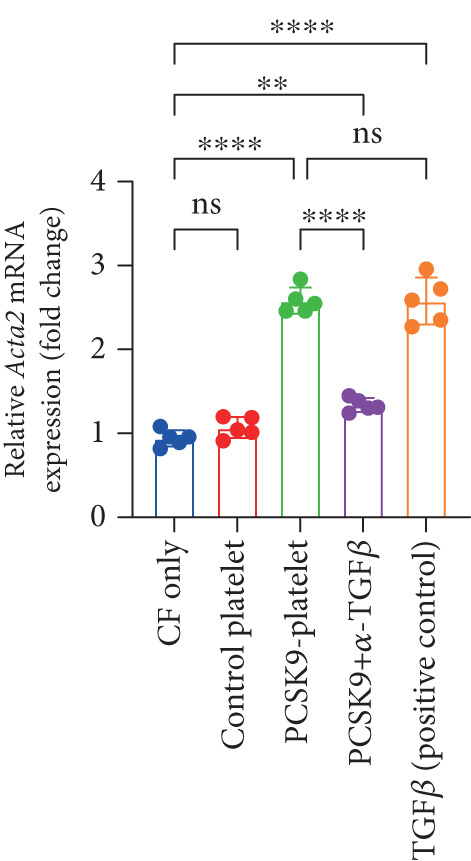
(h)

**Figure:**
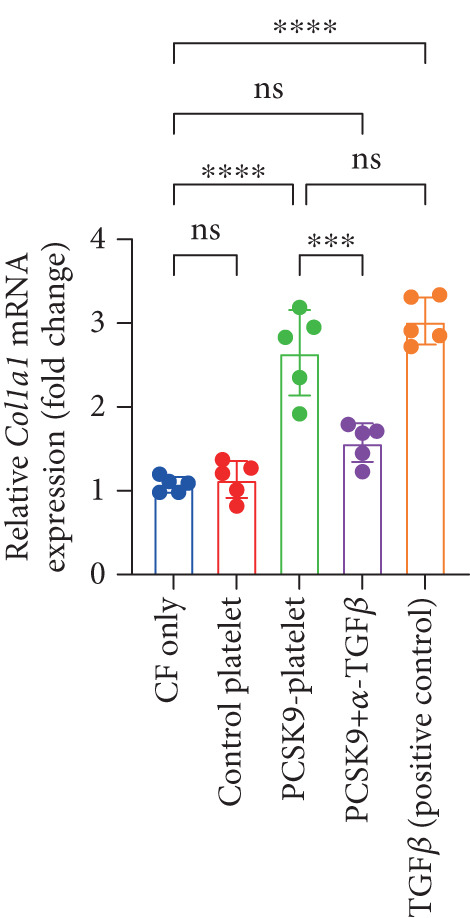
(i)

**Figure:**
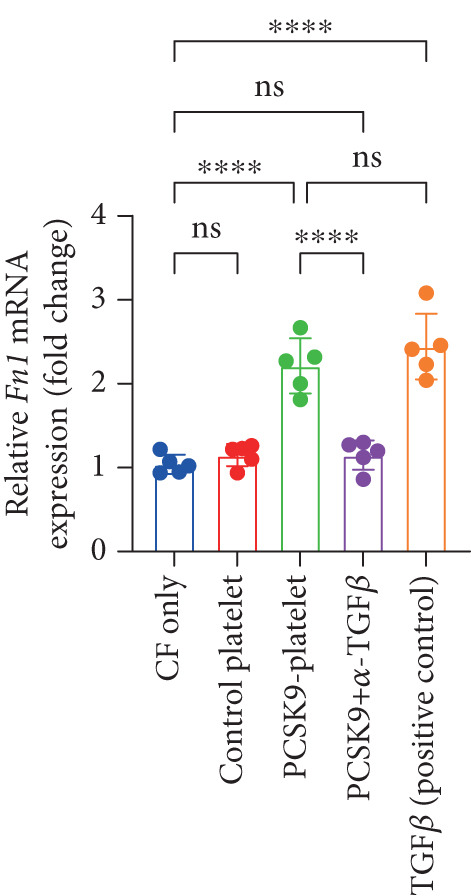
(j)

Immunofluorescence staining of fibroblasts was performed with DAPI (blue), vimentin (green), and *α*‐SMA (red). In the platelet coculture system, PCSK9 treatment significantly induced *α*‐SMA expression and cytoskeletal remodeling; in contrast, *α*‐SMA signaling was significantly reduced in the TGF‐*β* blockade group. Statistical analysis of *α*‐SMA‐associated immunofluorescence signal intensity revealed that the intensities were significantly greater in the PCSK9 group and the TGF‐*β*‐positive control group than in the other groups (Figure 5f,g *p* < 0.0001).

After the fibroblasts were cocultured with platelets from different treatment groups for 24 h, qPCR was used to detect the expression of fibrosis‐related genes. The results revealed that the expression levels of Acta2 (Figure 5h, *p* < 0.0001), Col1a1 (Figure 5i, *p* < 0.001), and Fn1 (Figure 5j, *p* < 0.001) were significantly upregulated in the PCSK9 group and that blockade of TGF‐*β* expression reversed this effect.

## 4. Discussion

Although many studies have elucidated the role of PCSK9 in the development of AMI through the alteration of plasma levels of LDL‐C via the PCSK9‐LDLR axis [21, 22], studies on its association with platelet activation‐induced AMI remain limited [13, 23]. Specifically, during the middle and late stages of AMI progression, the transition from early inflammatory activation to a fibrotic cascade aimed at repairing damaged myocardium eventually leads to scar formation. Some studies have shown that PCSK9 inhibitors reduce cardiac fibrosis in rats with reperfusion injury by inhibiting the inflammatory response. [24–26] However, the specific mechanism, particularly the origin of TGF‐*β*, remains unclear.

Platelets are defined as small, anucleated blood cells derived from proplatelets, a key intermediate structure. Proplatelets are specifically generated by polyploid megakaryocytes (MKs), which are primarily localized within the sinusoids of the bone marrow (BM), the primary hematopoietic organ [27]. Platelet adhesion and aggregation are not only essential for primary hemostasis at the site of vascular disease but also play key roles in the development of acute arterial thrombotic occlusion [28]. Pathology‐induced platelet activation, triggered by contact with subendothelial collagen in areas of atherosclerotic plaque erosion or rupture, reflects a major pathophysiological mechanism of AMI and ischemic stroke [29]. In addition to their roles in hemostasis and pathological arterial thrombosis, emerging evidence supports a critical role for platelets in inflammation, cell repair, and regeneration. Platelets are known to have important immune functions and are the largest reservoir of physiological TGF‐*β*1 [30, 31]. The TGF‐*β*1 content in platelets is 40–100 times greater than that in other cells. Itga2b is a key adhesion molecule on the platelet surface and is involved in platelet activation, aggregation, and cellular signal transduction [32]. After AMI onset, vascular endothelial injury serves as the “initial signal” triggering platelet responses; however, platelet activation, aggregation, and granule release depend on the functional activation of Itga2b [33]. A study by Qi et al. revealed that PCSK9 directly affects agonist‐induced platelet aggregation, dense granule ATP release, integrin *α*IIb*β*3 activation, *α*‐granule release, platelet spreading, and clot retraction [2].

Our study is the first to suggest that PCSK9 promotes postinfarction myocardial fibrosis by increasing the level of platelet‐derived TGF‐*β*. We constructed PCSK9 KO mice and evaluated differences in fibrosis severity between control and postinfarction mice. We found that PCSK9 deletion facilitates the repair of postinfarction myocardial fibrosis. Cellular sequencing analysis of the two groups of mice reported that platelet‐associated Itga2b is a key mediator through which PCSK9 affects post‐AMI myocardial fibrosis. Finally, we further verified—via intracellular, extracellular, and coculture experiments—that PCSK9 promotes platelet TGF‐*β* secretion and modulates the transformation of cardiac fibroblasts into myofibroblasts. These findings confirm that PCSK9 promotes myocardial fibrosis by increasing the level of platelet‐derived TGF‐*β*. After AMI onset, platelets are activated via the Itga2b‐mediated adhesion–aggregation process. Proteases in the local microenvironment subsequently convert TGF‐*β* into its active mature form. Mature TGF‐*β* exerts its effects by binding to TGF‐*β* receptors (T*β*Rs) on the surface of target cells, activating two key downstream signaling pathways: the Smad‐dependent pathway and the Smad‐independent pathway. These pathways increase fibroblast proliferation and migration, inhibit the activity of collagenases (e.g., MMPs), and reduce collagen degradation. Through their synergistic action, the two pathways collectively drive the development and progression of myocardial fibrosis.

The timely and spatial suppression of postinfarction inflammatory responses depends on two factors: the release of secreted anti‐inflammatory mediators (e.g., IL‐10, members of the TGF‐*β* family, and lipid‐derived procatabolic mediators) and the activation of intracellular “STOP signals” that suppress innate immune responses [34]. Previous studies have shown that the TGF‐*β*/Smad signaling pathway plays a key role in tissue repair [35]. TGF‐*β*1 promotes the transformation of cardiac fibroblasts into myofibroblasts; *α*‐SMA, an indicator of mature myofibroblasts, is significantly upregulated in the infarct border zone [36]. Myofibroblasts further release proinflammatory factors, angiotensin II, and other fibrosis‐promoting cytokines, exacerbating cardiac fibrosis [37]. TGF‐*β* is abundant in the bone and platelets; during AMI, platelets infiltrate the ischemic myocardium and significantly modulate the surrounding microenvironment via proinflammatory and anti‐inflammatory mediators [38]. However, few studies have investigated the effects of platelet‐derived TGF‐*β* on the myocardial microenvironment and related inflammatory immune cells after AMI. Building on previous studies showing that PCSK9 enhances platelet activation, in vivo thrombosis, and post‐AMI myocardial.

infarction expansion [2], this study further explored the effect of PCSK9 on fibroblast‐induced myocardial fibrosis by sequencing analysis. This work expands our understanding of the mechanism by which PCSK9 acts on myocardial tissueafter AMI and provides a theoretical basis for the role of PCSK9 inhibitors inpost‐AMI myocardial repair.

This study has several limitations. First, we used PCSK9 KO mice but lacked validation with PCSK9 agonists or viral overexpression. Second, lipid changes and their relationship with fibrosis were not monitored simultaneously.

## 5. Conclusion

PCSK9 promotes platelet activation, induces the secretion of platelet‐derived TGF‐*β*, and thereby accelerates myocardial fibrosis after MI.

**Nomenclature**
PCSK9: proprotein convertase subtilisin/Kexin 9
LDLR: low‐density lipoprotein receptor
LDL‐C: low‐density lipoprotein cholesterol
ECM: extracellular matrix
LVEF: ejection fraction
LVID: d(LV end‐diastolic diameter)
LVID: s(LV end‐systolic diameter)

## Ethics Statement

Animal experiments were executed with the approval of IACUC, Bojin Biotechnology.

## Disclosure

All authors approved the final manuscript and agreed to publication in Human Mutation.

## Conflicts of Interest

The authors declare no conflicts of interest.

## Author Contributions

Feifei Wang designed the study. Qianyun Wang and Wenxiang Huang performed the experiments and analyzed the data together. Dianmin Xia drafted the manuscript. All authors critically revised it.

## Funding

This work was supported by the Science and Technology Projects in Guangzhou, 2023A03J0563.

## Data Availability

Any materials can be obtained from the authors upon request.
